# Acute pancreatitis during valproic acid administration in a patient with vascular dementia, epileptic seizures, and psychiatric symptoms: a case report

**DOI:** 10.1186/s13256-023-03964-4

**Published:** 2023-05-29

**Authors:** Mako Yanaga, Naomichi Okamoto, Reia Hashimoto, Ryohei Igata, Yuki Konishi, Atsuko Ikenouchi, Naoki Isomoto, Takahiro Shinkai, Masaru Harada, Reiji Yoshimura

**Affiliations:** 1 Department of Psychiatry, University of Occupational and Environmental Health, Fukuoka, 8078555 Japan; 2 Medical Center for Dementia, Hospital of the University of Occupational and Environmental Health, Fukuoka, 8078555 Japan; 3 Third Department of Internal Medicine, University of Occupational and Environmental Health, Fukuoka, 8078555 Japan

**Keywords:** Valproic acid, Acute pancreatitis, Vascular dementia, Symptomatic epilepsy, Psychiatric symptoms

## Abstract

**Background:**

Valproic acid (VPA) is a relatively safe drug widely used for the treatment of epileptic seizures and mania in bipolar disorder, as well as the prevention of migraine headaches. Here, we present a case of VPA-induced pancreatitis in a patient with vascular dementia, epileptic seizures, and psychiatric symptoms. He had no distinctive abdominal symptoms.

**Case presentation:**

A 66-year-old Japanese man was treated with VPA for agitation and violent behavior due to vascular dementia, epileptic seizures, and psychiatric symptoms. During admission, he experienced a sudden decrease in consciousness and blood pressure. Abdominal findings were unremarkable; however, blood tests showed an inflammatory response and elevated amylase levels. Contrast-enhanced abdominal computed tomography showed diffuse pancreatic enlargement and inflammation extending to the subrenal pole. VPA-induced acute pancreatitis was diagnosed, VPA was discontinued, and high-dose infusions were administered. Acute pancreatitis resolved after treatment initiation.

**Conclusions:**

Clinicians should be aware of this relatively rare side effect of VPA. Diagnosis may be challenging in elderly people and patients with dementia as they may present with non-specific symptoms. Clinicians should consider the risk of acute pancreatitis when using VPA in patients who cannot report spontaneous symptoms. Blood amylase and other parameters should be measured accordingly.

## Background

Valproic acid (VPA) is a widely used drug for the treatment of epileptic seizures and mania in bipolar disorder, as well as for the prevention of migraine headaches [1]. VPA is a relatively safe drug [2]; however, a rare side effect is acute pancreatitis, with a frequency of approximately 0.003–0.7% [3]. VPA-induced pancreatitis is more common in young patients than in elderly patients [4]. While one report showed that the prognosis was worse in half of the cases [5], another reported a relatively good prognosis [4]; therefore, the outcome may vary from case to case.

Here, we present a case of VPA-induced pancreatitis in a patient with vascular dementia, epileptic seizures, and psychiatric symptoms. In this case, a sudden loss of consciousness and hypotension occurred without abdominal symptoms, including pain.

## Case presentation

We report the case of a 66-year-old Japanese man. He developed a subarachnoid hemorrhage at the age of 56 (10 years ago). After the subarachnoid hemorrhage, he experienced cognitive decline and generalized epileptic seizures. He received levetiracetam 1000 mg/day, carbamazepine 400 mg/day, and VPA 400 mg/day to control generalized epileptic seizures. Prior to referral to the hospital, the patient was wheelchair-bound and required assistance with bathing and toileting; however, he could communicate briefly. He had no history of alcohol use or smoking.

**Fig. 1:**
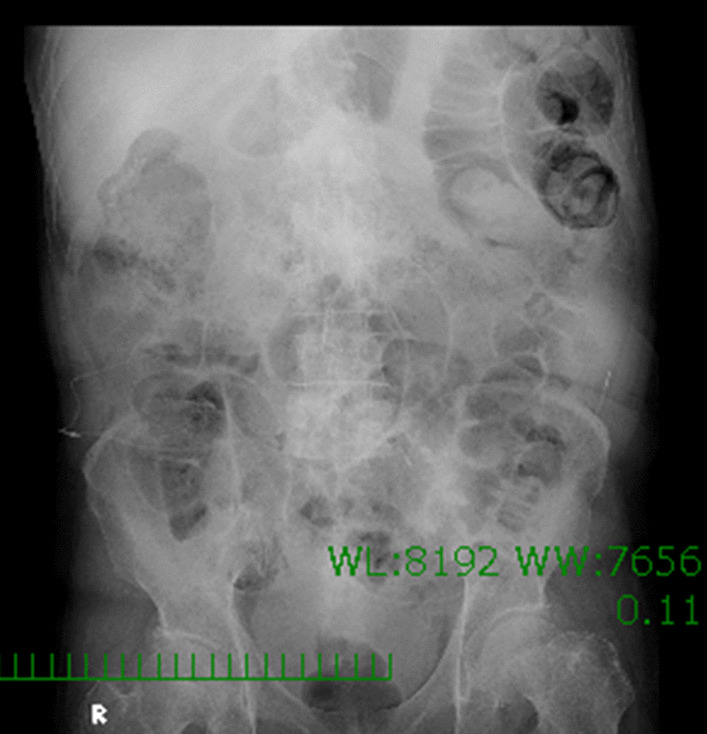
Simple abdominal radiography. Simple abdominal radiography shows gas and stool retention in the intestinal tract; however, intestinal obstruction is negative

On April 19, he was referred to our hospital for medication adjustment due to the sudden onset of agitation and violent behavior. On admission, his vital signs were normal (temperature 36.5 °C, heart rate pulse 60 bpm, blood pressure 138/95 mmHg). In addition to the above drugs, he was periodically taking antihypertensive drugs (telmisartan 60 mg/day, enalapril 5 mg/day, cilnidipine 10 mg/day), and psychotropic drugs (quetiapine 125 mg/day, tiapride 100 mg/day, ramelteon 8 mg/day), which have been added 6 months prior to our referral. The patient’s psychiatric symptoms included intermittent episodes of irritability and agitation. He experienced sudden somnolence, but there were no abnormal neurological findings such as involuntary movements or seizure. Levetiracetam had been administered at the dose of 1000 mg/day for a long time before the onset of agitation and violent behavior; therefore, we denied increased irritability as a drug side effect. Although there were no epileptic waves evident on the electroencephalogram, we suspected epileptic seizure with interictal psychiatric symptoms and increased the dose of VPA from 400 to 800 mg/day on May 7 to control irritability and agitation. There were no changes in the other prescribed medications. After the dose was increased, there was still no improvement in the patient's irritability and violent behavior; therefore, the dose of VPA was increased further to 1200 mg/day on May 14. As liver enzyme elevations (aspartate aminotransferase: 47 U/I, alanine aminotransferase: 108 U/I) were observed with an increased dose, the dose of VPA was reduced to 800 mg on May 21, and his liver enzyme levels improved (aspartate aminotransferase: 28 U/I; alanine aminotransferase: 66 U/I). He presented with complaints of nausea and mild headache on May 22. The patient had no complaints of spontaneous and objective abdominal symptoms. Simple abdominal radiography showed gas and stool retention in the intestinal tract; however, intestinal obstruction was negative (Fig. 1). A brain computed tomography (CT) scan showed no evidence of cerebral hemorrhage or other new lesions.

**Fig. 2:**
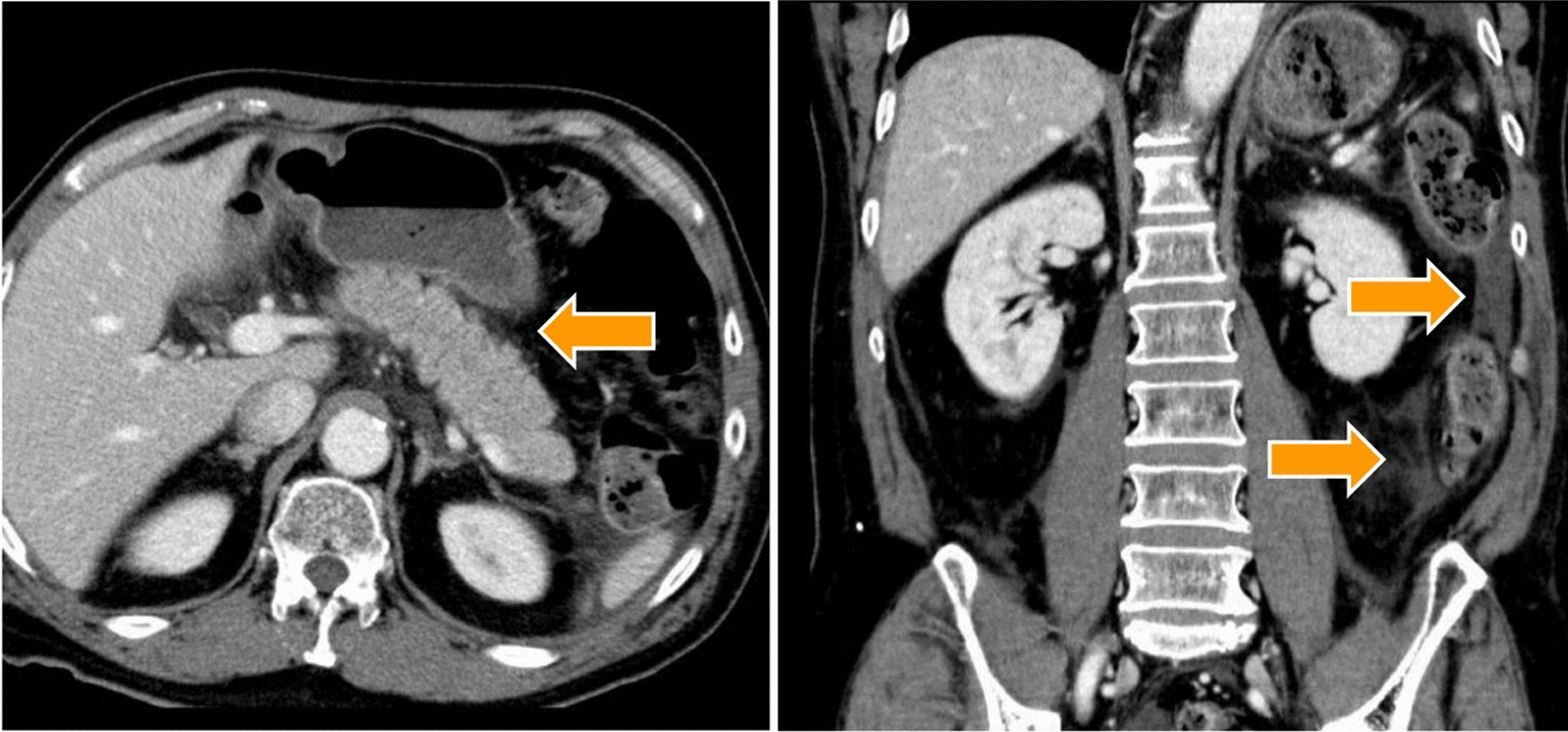
Contrast-enhanced abdominal computed tomography scan. Contrast-enhanced abdominal computed tomography scan shows diffuse pancreatic enlargement (arrow on axial plane) and inflammation extending beyond the sub-renal pole and beyond (arrow on coronal plane). There is no contrast failure zone, suggesting contrast computed tomography grade 2 acute pancreatitis

The patient had a sudden decrease in level of consciousness and blood pressure (temperature 36.1 °C, heart rate pulse 79 bpm, blood pressure 54/38 mmHg) on May 23, with poor physical findings, including abdominal findings and normal head CT, electrocardiogram, and echocardiogram findings. Blood tests showed an inflammatory reaction (white blood cell: 6.3 × 10^3^/µL, neutrophil: 91.6%, C-reactive protein: 4.97 mg/dL) and elevated amylase (1926 U/I). Contrast-enhanced abdominal CT showed diffuse pancreatic enlargement (arrow on axial plane in Fig. 2) and inflammation extending beyond the subrenal pole (arrow on coronal plane in Fig. 2). There was no contrast failure zone, suggesting contrast CT grade 2 acute pancreatitis.

**Fig. 3:**
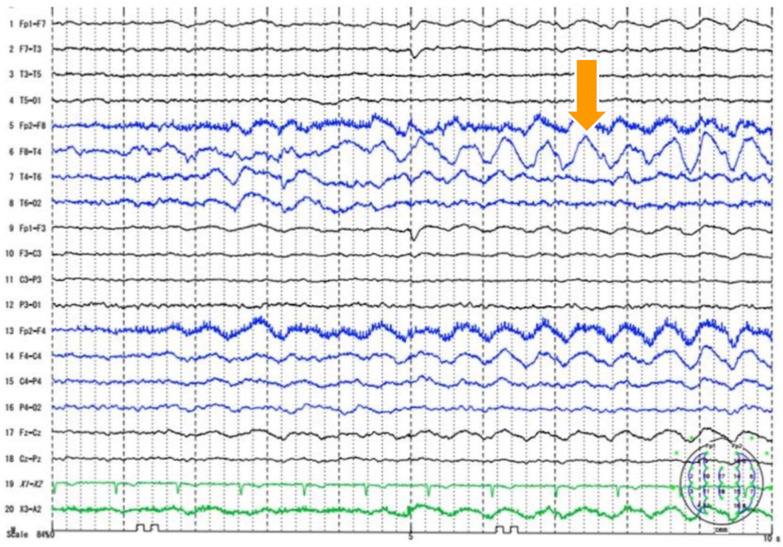
An electroencephalogram. An electroencephalogram shows right frontal rhythmic delta waves (arrow), with findings suggestive of epileptic seizures

**Fig. 4:**
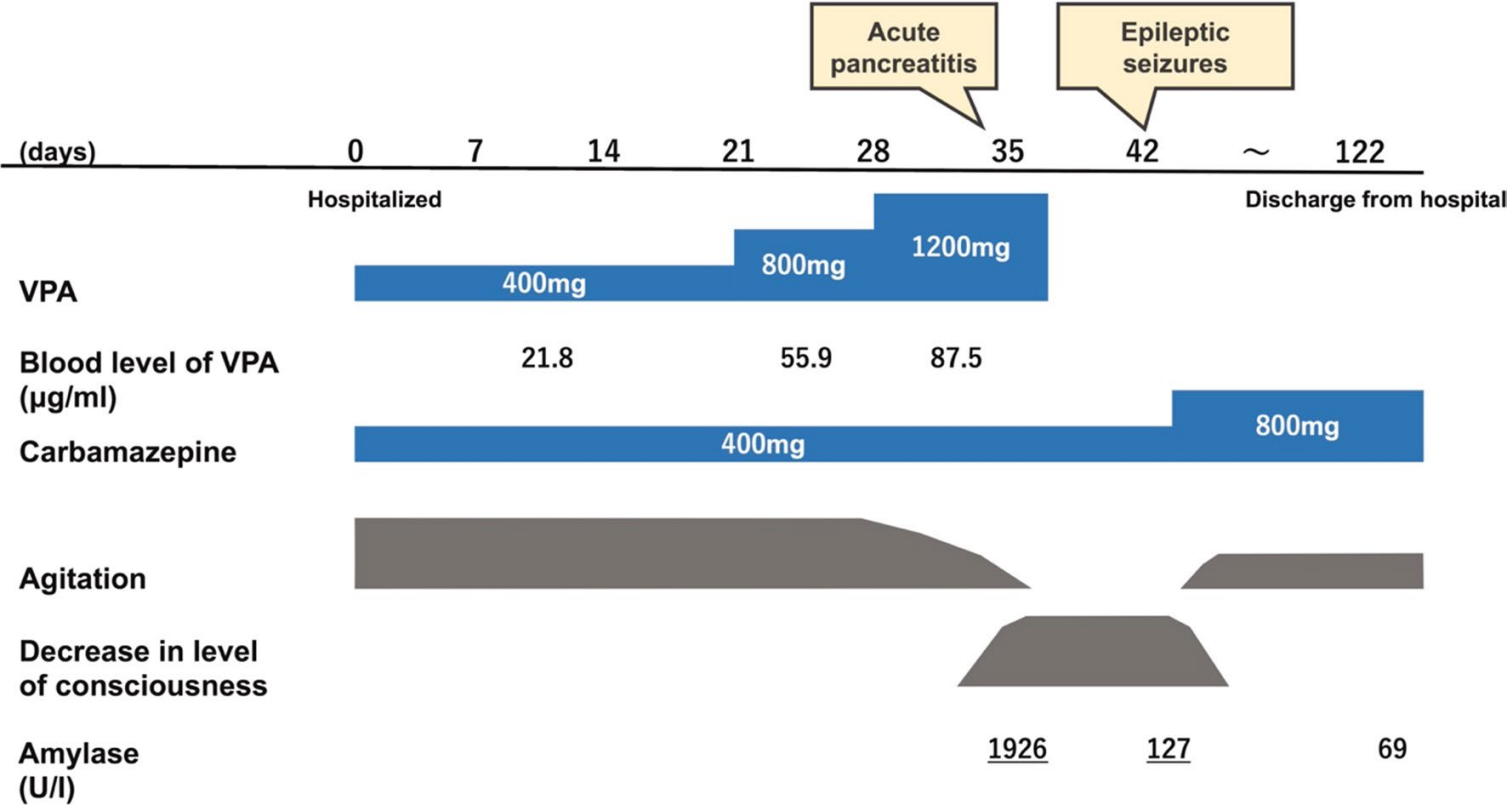
Disease course summary. We show patient’s disease course summary

The patient had undergone cholecystectomy for cholecystitis at the age of 61 (5 years ago) and was not hyperlipidemic. We discontinued VPA and initiated treatment with a high-volume infusion since we suspected drug-induced acute pancreatitis. The inflammatory response and elevated amylase enzyme levels improved, and the acute pancreatitis resolved after the treatment. We diagnosed VPA as the suspected drug based on three items of the World Health Organization–Uppsala Monitoring Centre Causality Rating Scale (e.g., event or laboratory test abnormality, with reasonable time relationship to drug intake and unlikely to be attributed to disease or other drugs, response to withdrawal clinically reasonable) [6]. He had somnolence after discontinuation of VPA on May 31. An electroencephalogram showed right frontal rhythmic delta waves with findings suggestive of epileptic seizures (arrow in Fig. 3). Therefore, we made a definitive diagnosis of epileptic seizures with psychiatric symptoms. We increased the dose of carbamazepine from 400 to 800 mg for epileptic seizure control. After increasing the dose of carbamazepine, his agitation and violent behavior decreased, and he was discharged from our hospital on August 18, and admitted to a facility (blood data and disease course summary are shown in Table 1 and Fig. 4). At the 6-month follow-up, psychiatric symptoms were stable and there was no recurrence of pancreatitis.

**Table 1:** Patient’s blood data change

	April 19 (hospitalized)	May 19	May 23 (acute pancreatitis)	May 25	June 1	August 3 (discharge from hospital)
WBC, cells/μL	3200	3400	6300	7200	5500	3100
RBC, × 10^6^ cells/μL	3.87	3.65	3.80	3.30	3.04	3.87
PLT, × 10^3^ cells/μL	162	196	110	92	370	183
AST, IU/L	42	47	19	14	22	25
ALT, IU/L	55	108	46	30	33	53
Amylase, IU/L	–	–	1926	662	127	–
BUN, mg/dL	12	14	32	11	5	12
Creatinine, mL/min	0.89	0.85	1.85	0.82	0.65	0.81
CRP, mg/L	0.30	–	4.97	9.83	10.1	0.16
Blood level of VPA, μg/mL	–	87.5	–	–	–	–
Blood level of carbamazepine, μg/mL	–	–	–	7.8	9.7	–

*ALT* alanine aminotransferase, *AST* aspartate aminotransferase, *BUN* blood urea nitrogen, *CRP* C-reactive protein, *PLT* platelet, *RBC* red blood cell, *VPA* valproic acid, *WBC* white blood cell

## Discussion

Here, we presented a case of VPA-induced severe acute pancreatitis with vascular dementia, epileptic seizures, and psychiatric symptoms. This report is clinically significant because (1) clinicians should be aware of this relatively rare adverse effect, and (2) VPA-induced acute pancreatitis is predominant in younger patients and is rarely reported in elderly patients, and diagnosis can be difficult as there are fewer complaints in the elderly and patients with dementia.

Acute pancreatitis, an extremely rare side effect of VPA, was first noted in two case reports in 1979 [7, 8], and its frequency is approximately 0.003–0.7% [3]. The pathophysiology of VPA-induced pancreatitis is still not well known [4]; however, it is thought to be due to pancreatic duct contraction, cytotoxic and metabolic effects, accumulation of toxic metabolites or intermediates, and hypersensitivity reactions [9]. It is speculated that glutathione peroxidase and selenium are reduced in cases of VPA-induced acute pancreatitis, and that free radicals injure pancreatic cell membranes due to reduced antioxidant activity [10]. In this case, VPA-induced acute pancreatitis occurred after VPA was increased from 400 to 1200 mg/day (which was subsequently reduced to 800 mg/day due to liver damage); however, there was no relationship between the occurrence of VPA-induced acute pancreatitis and blood VPA concentration, and it could occur at any time after the onset of therapy [11, 12].

Presentations of pancreatitis include epigastric or diffuse abdominal pain (80–95%), nausea and vomiting (40–80%), abdominal distension, fever, breathlessness, impaired consciousness, with pyrexia, low oxygen saturation, tachypnoea, tachycardia, hypotension, abdominal guarding, ileus and/or oliguria [13]. A diagnosis of acute pancreatitis requires two out of three criteria: (1) abdominal pain consistent with pancreatitis, (2) serum amylase or lipase levels three or more times higher than the upper limit of normal, and (3) findings consistent with pancreatitis on cross-sectional abdominal imaging [13]. Diagnosing drug-induced pancreatitis requires ruling out more common etiologies such as gallstone pancreatitis and ethanol-induced acute pancreatitis [14]. If the pancreatitis resolves after discontinuation of a drug with the potential to cause pancreatitis, suspicion of drug-induced pancreatitis is increased [14]. Diagnosis of VPA-induced acute pancreatitis can be difficult because there are less complaints in elderly people and patients with dementia. VPA-induced acute pancreatitis is predominant in younger patients with a mean age of 12.7 years [4] and is rarely reported in elderly patients over 60 years, as in this case. The current patient presented with symptoms of shock due to a decrease in blood pressure, which led to early detection and therapeutic intervention. Oxygen, intravenous fluid resuscitation, analgesia and nutrition are fundamentals of initial treatment [13]. The subsequent prognosis in this case was good; however, the patient did not exhibit early abdominal pain symptoms, which are commonly reported. Clinicians should consider the risk of acute pancreatitis when VPA is used in patients who cannot complain of spontaneous symptoms, such as patients with dementia, and should measure blood amylase and other parameters as necessary.

## Conclusions

In conclusion, VPA-induced acute pancreatitis may occur in elderly individuals and patients with dementia. Furthermore, clinicians should be mindful of delayed diagnosis because of the lack of subjective symptoms. Blood amylase and other parameters should be measured as necessary.

## Data Availability

The datasets used and/or analyzed during the current case report are available from the corresponding author upon reasonable request.
